# Comparative Study of the Diagnostic Value of Panoramic and Conventional Radiography of the Wrist in Scaphoid Fractures

**DOI:** 10.5812/iranjradiol.10099

**Published:** 2012-12-27

**Authors:** Fatemeh Ezoddini Ardakani, Maryam Zangoie Booshehri, Seyed Hossein Saeed Banadaki, Reza Nafisi-Moghadam

**Affiliations:** 1 Department of Oral and Maxillofacial Radiology, Faculty of Dentistry, Shahid Sadoughi University of Medical Sciences, Yazd, Iran; 2 Department of Orthopedics, Faculty of Medicine, Shahid Sadoughi University of Medical Sciences, Yazd, Iran; 3 Department of Radiology, Faculty of Medicine, Shahid Sadoughi University of Medical Sciences, Yazd, Iran

**Keywords:** Scaphoid Bone, Fractures, Bone, Radiography, Wrist, Radiography, Panoramic

## Abstract

**Background:**

Scaphoid fractures are the most common type of carpal fractures.

**Objectives:**

The aim of the study was to compare the diagnostic value of panoramic and conventional radiographs of the wrist in scaphoid fractures.

**Patients and Methods:**

The panoramic and conventional radiographs of 122 patients with acute and chronic wrist trauma were studied. The radiographs were analyzed and examined by two independent radiologist observers; one physician radiologist and one maxillofacial radiologist. The final diagnosis was made by an orthopedic specialist. Kappa test was used for statistical calculations, inter- and intra-observer agreement and correlation between the two techniques.

**Results:**

Wrist panoramic radiography was more accurate than conventional radiography for ruling out scaphoid fractures. There was an agreement in 85% or more of the cases. Agreement values were higher with better inter and intra observer agreement for panoramic examinations than conventional radiographic examinations.

**Conclusion:**

The panoramic examination of the wrist is a useful technique for the diagnosis and follow-up of scaphoid fractures. Its use is recommended as a complement to conventional radiography in cases with inconclusive findings.

## 1. Background

Scaphoid fractures are the most common carpal fractures (1). Their diagnosis could be difficult or even missed. Conventional radiographs are the most common method for clinical evaluation of suspicious scaphoid fractures. In cases of suspicious fracture of the scaphoid with negative radiological findings, the wrist is usually fixed and immobilized. The radiograph is repeated after 10-12 days. Diagnosis of scaphoid fracture is important in order to prevent complications such as delayed union, nonunion, avascular necrosis and osteoarthritis, especially when the diagnosis is missed initially and the time allocated for treatment is insufficient (1-3). In cases with a clinical possibility of scaphoid fracture in which radiological findings are negative or equivocal, various radiographical methods are presently used in order to increase the accuracy of diagnosis. These methods include bone scintigraphy (4), CT scan (5), MRI (6) and ultrasonography (7). Panoramic technique or orthopantomography is widely used in evaluation of the dentomaxillofacial region. In 1991, the first article regarding panoramic carpal radiography (8) and in another article (9), panoramic radiography was reported to be a useful supplement for conventional radiography in the evaluation of scaphoid fractures. Diagnosis of fractures and non-unions in patients who were evaluated with panoramic radiographs of the wrist after conventional radiographs showed a significant improvement.

Accurate and appropriate treatment promises the best prognosis. Radiography is useful not only for early diagnosis, but also for controling the quality of treatment and healing of fractures, so that unstable fractures can be identified (10). Diagnosis of stability of the fracture is made by conventional radiography and if necessary, CT scan accurately shows the presence and severity of displacement (11). There are many factors that are responsible for delayed union or nonunion of the scaphoid bone; for example nonunion is more common in the middle and proximal portion of the scaphoid bone. These factors include inappropriate blood supply in the affected area, delayed diagnosis, anatomical location of the fracture and the severity of displacement (12).

## 2. Objectives

In the present study, we evaluate the role of wrist panoramic radiography in the analysis of wrist trauma in comparison with conventional wrist radiography.

## 3. Patients and Methods

### 3.1. Patient Selection

This study carried out on 62 left and 60 right wrists in patients aged between 10 and 70 years with wrist trauma referred to the orthopedist with indications for radiography of the wrist. On referral to the radiology clinic, conventional radiographies of the wrist were performed and the radiologist recorded the presence or absence of fracture in a special form. Then the process of the wrist panoramic radiography was explained to all the participants in detail and after obtaining written consent, wrist panoramic radiography was taken.

Follow up radiographies were carried out after 2 to 19 months. A total of three cases with scaphoid fractures that had undergone surgical treatment were excluded from the study. The panoramic radiography of these three cases was not satisfactory because of severe limitation in movement. The study was approved by the Ethics Committee of Shahid Sadoughi University of Medical Sciences.

### 3.2. Imaging Technique and Interpretation

The wrist radiographs were taken with the help of special tools that properly positioned and immobilized the hands. The patients were in the standing, sitting or sleeping positions. The panoramic radiographs were obtained in the standard posteroanterior position with the wrist in the sagittal plane parallel to the sagittal sinus of the patient. The standard posteroanterior panoramic radiograph was obtained by approximately 10 degree rotation of the wrist and 60-64 kW and four to six mA. The period of rotation was approximately 12 seconds. Patients with a history of wrist surgery were excluded from the study. The examiners were two radiologists and an orthopedist. The panoramic radiographs were examined by a maxillofacial radiologist, while the conventional radiographs were examined by a radiologist. The patients were divided into two groups of acute trauma and chronic phase. The protocol of conventional radiography included four projections (posteroanterior, lateral, oblique in pronation and ulnar rotation), while the panoramic radiography was obtained in the posteroanterior view. The panoramic radiograph was taken by a Planmeca 2002 EC Proline machine with 80 kVp, 12 mA and 15 seconds. Digital images were obtained by a Japanese made Konica Computed Radiography machine. All of the images were taken by one machine and technician. The films and printer used was Konica. The primary diagnosis was made by a radiologist. The following diagnostic criteria were considered on the basis of various studies;

1. Negative radiography: absence of scaphoid fracture

2. Fracture union: presence of trabeculations or bony bridge on the line of fracture.

3. Suspected fracture union: absence of radiological signs of fracture union.

4. Fracture nonunion: presence of clear gap in the fracture region more than five months after trauma or injury and included two subdivisions.

A questionnaire was designed for diagnosis of the radiographies and filled in according to the mentioned classification. Initially, the orthopedist examined the patients and requested a wrist radiography. The radiographs were studied by the orthopedist and the radiological diagnosis was recorded in the questionnaire. Then, after explanation by the orthopedist and referral to the maxillofacial radiology clinic, a panoramic wrist radiograph was obtained.

The classification of the images was recorded in the questionnaires by consensus between the orthopedist and the maxillofacial radiologist. Of the total of 122 patients, 40 conventional radiographs and 40 panoramic radiographs were selected randomly and placed in numbered envelopes. The evaluation of these patients was done by three observers (one orthopedist with 20 years of experience and two radiologists with 25 years of experience). None of the observers had access to previous information of the selected radiographs or panoramic technique.

We considered the orthopedist’s opinion for diagnosis of fracture as gold standard and compared the accuracy of conventional and panoramic radiography with it. The agreement of the two radiologic methods, conventional and panoramic, was evaluated as well. We used STARD method (checklist and diagram) to write the manuscript (13).

### 3.3. Statistical Analysis

SPSS 16 for Windows (SPSS Inc., Chicago, Illinois, USA) was used for statistical analysis. The agreement of conventional and panoramic radiographies was determined by Kappa coefficient. Kappa coefficient was used to study inter- and intra-observer agreement by comparing levels of agreement between the two techniques. A kappa value of 0.20 or less indicates slight, 0.21-0.40 fair, 0.41-0.60 moderate, 0.61-0.80 good and 0.81-1.00 shows very good agreement. All diagnostic indices were reported with 95% Confidence Interval. A p-value less than 0.05 was considered as significant.

## 4. Results

A total of 122 patients with suspected scaphoid fracture who were referred for wrist radiography from 2008 till 2010 were included in the study. The mean age of the patients was 33.63±14.8 years with a range of 10 to 69 years. Sixty-six of the patients (54.1%) were men and 56 (45.9%) were women. The results of the three techniques of diagnosis of scaphoid fracture including conventional radiography, panoramic radiography and diagnosis of the treating specialist on the basis of results of surgery, radiographies and follow up till the final result that was considered as the final diagnosis in the study were determined for all of the acute cases, but the results of the cases with chronic scaphoid fractures were followed in only 37 cases with regards to healing of fractures.

Totally, 81 cases (66.4%) had scaphoid fractures, 41 cases (33.6%) did not have a scaphoid fracture and 28 cases (75.7%) had healed fractures while the rest (24.3%) had nonunion fractures. The results of conventional radiographies and panoramic radiographies and their comparison with the final diagnosis are shown in Table 1. All the results were reported with a 95% Confidence Interval.

**Table 1:** Diagnostic Indices of Conventional and Panoramic Radiography in Addition to the Agreement of These Diagnostic Methods with Gold Standard in Scaphoid Fracture

	TP	FN	TN	FP	Sen. [95% CI]	Spec. [95% CI]	PPV [95% CI]	NPV [95% CI]	Accuracy [95% CI]	Kappa [95% CI]	P-Value
Conventional Radiography	60	21	7	34	74.1% [64.6- 83.6]	17.1% [5.6- 28.6]	63.8% [54.1- 73.5]	25% [9- 41]	54.9%. [46.1-63.7]	-0.096 [-0.26-0.066]	0.272
Panoramic Radiography	78	3	36	5	96.3% [92.2- 100]	87.8% [77.8- 97.8]	94% [88.9- 99.1]	92.3% [84- 100]	93.4% [89-97.8]	0.84 [0.75- 0.95]	<0.0001

Abbreviations: TP, True positive; FN, False negative; TN, True negative; FP, False positive; Sen., Sensitivity; Spec., Specificity; PPV, Positive predictive value; NPV, Negative predictive value; CI, Confidence interval

The relation between the results of conventional radiography and the gold standard was studied by Kappa test and a Kappa coefficient of -0.096 was obtained (P-value = 0.272). There was no significant relationship between the conventional radiography and the gold standard. Conventional radiography diagnosed only 74% of the wrist scaphoid fractures and was not able to confirm all cases without fractures.

The relation between the results of panoramic radiography and the gold standard was studied by Kappa test and a coefficient of 0.851 was obtained (P-value<0.001). In order to compare the sensitivity between the two methods of conventional and panoramic radiography in diagnosis of scaphoid fractures, Z-test was used and with a P value of 0.01, the sensitivity of conventional radiography was significantly lower than that of panoramic radiography. Similarly, the specificity of panoramic radiography were significantly better than that of conventional radiography (P value < 0.001). Besides, the positive and negative predictive values and accuracy of panoramic radiography was significantly better than that of conventional radiography (P < 0.001 in all of the cases).

The calculated diagnostic values in both genders was approximately the same and similar to the overall diagnostic values. The relation between the results of conventional radiography and the final diagnosis in both men and women with P-values of 0.727 and 0.22 was not significant.

All the diagnostic indices of panoramic radiography in both genders were higher than 85% that depicts the high diagnostic value of the technique in comparison to the final diagnosis. The diagnostic value of panoramic radiography was higher in women than that in men. The agreement between the panoramic radiography and the gold standard was studied by Kappa test and in both genders, this agreement was significant (both P-values<0.001). The results of conventional radiography in the diagnosis of scaphoid fractures on the basis of age are shown in Table 2.

**Table 2:** Diagnostic Indices of Conventional and Panoramic Radiography in Addition to the Agreement of These Diagnostic Methods with Gold Standard in Scaphoid Fracture in Terms of Different Age Groups

		TP ^a^	FN ^a^	TN ^a^	FP ^a^	Sen. ^a^ [95% CI ^a^]	Spec. ^a^ [95% CI]	PPV ^a^ [95% CI]	NPV ^a^ [95% CI]	Accuracy [95% CI]	Kappa [95% CI]	P-Value
Conventional Radiography	10-34 (y)	35	9	3	20	79.5% [67.6 – 91.4]	13% [<1- 26.7)	63.6% [50.9-76.3]	25% [0.5- 49.5]	56.7% [44.9- 68.5]	-0.08 [-0.28-0.12]	0.453
	35-69 (y)	25	12	4	14	67.6% [52.5 – 82.7]	22.2% [31-41.4]	64.1% [49.1-79.1]	25% [3.8-46.2]	52.7% [39.5- 65.9]	-0.11 [-0.36-0.15]	0.434
Panoramic Radiography	10-34 (y)	44	0	21	2	100% [91-100]	91.3% [79.8-100]	95.7% [89.9-100]	100% [83-100]	97% [92.7- 100]	0.93 [0.84-1]	<0.0001
	35-69 (y)	34	3	15	3	91.9% [83.1- 100]	83.3% [66.1- 100]	91.9% [83.1-100]	83.3% [66.1- 100]	89.1% [80.9- 97.3]	0.75 [0.57- 0.94]	<0.0001

Abbreviations: TP, True positive; FN, False negative; TN, True negative; FP, False positive; Sen., Sensitivity; Spec., Specificity; PPV, Positive predictive value; NPV, Negative predictive value; CI, Confidence interval

The calculated diagnostic value in both age groups was approximately the same, while the relation between the conventional radiographical findings and final diagnosis in both groups was not significant (P > 0.5).

Panoramic radiography results in the diagnosis of wrist scaphoid fractures on the basis of age are shown in Table 2. The relation of panoramic radiography findings with the final results in the diagnosis of acute wrist scaphoid fractures in both age groups was significant (P < 0.001).

Conventional and panoramic radiography results in the diagnosis of chronic phase healing of scaphoid fractures are shown in Tables 3 and 4.

**Table 3:** Cross Tabulation of the Conventional Radiography Results Versus Gold Standard in the Diagnosis of Non Healing of Scaphoid Fracture in Chronic Setting

		Final Results		
		Non Healed No.[%]	Healed No.[%]	Total No.[%]
Conventional Radiography Results	Non Healed No.[%]	0[0]	12[42.9]	12[32.4]
	Healed No.[%]	0[0]	16[57.1]	16[43.2]
	Suspected Healed No.[%]	9[100]	0[0]	9[24.4]
	Total No.[%]	9[24.3]	28[75.7]	37[100]

**Table 4:** Diagnostic Indices of Panoramic Radiography for Non Healing of Scaphoid Fractures in Addition to Agreement of This Modality with Gold Standard in Chronic Setting

	TP ^a^	FN ^a^	TN ^a^	FP ^a^	Sen. ^a^ [95% CI ^a^]	Spec. ^a^ [95% CI]	PPV ^a^ [95% CI]	NPV ^a^ [95% CI]	Accuracy [95% CI]	Kappa [95% CI]	P-Value
Panoramic Radiography	9	0	16	12	100% [66-100]	57.1% [38.8-75.4]	42.8 [21.7-63.9]	100% [79-100]	67.6% [52.5– 82.7]	0.39 [0.17- 0.62]	0.002

Abbreviations: TP, True positive; FN, False negative; TN, True negative; FP, False positive; Sen., Sensitivity; Spec., Specificity; PPV, Positive predictive value; NPV, Negative predictive value; CI, Confidence interval

We had 9 cases of suspected healed patients on the conventional radiographs (Non healed on the table 4). On the whole, a total of 37 cases were followed in the chronic phase and the final results of healed scaphoid fractures were recorded.

Panoramic radiography did not have any suspicious or inappropriate results and was therefore better than conventional radiography. In this phase, the other diagnostic criteria of panoramic radiography were good and acceptable. The relation of the panoramic radiography results and final diagnosis was examined by Kappa test and with a P value of less than 0.001, the relation was meaningful, which means that panoramic radiography can be used in the follow up of scaphoid fracture healing.

In the chronic phase too, the results of conventional and panoramic radiography were compared with the final diagnosis based on age and gender. In contrast to panoramic radiography, conventional radiography did not have a significant relationship with the final diagnosis in different age and gender groups. The diagnostic values were calculated as follows: for men, the sensitivity, specificity, PPV, NPV and accuracy was 75%, 100%, 100%, 62.2% and 82.4%, respectively. These measures were 75%, 100%, 100%, 50% and 80% for women, respectively.

There was significant relationship between the results of panoramic radiography and final results in both genders (P < 0.001). The diagnostic value of panoramic radiography was the same in both genders.

The diagnostic values in the two age groups were calculated as follows: the sensitivity, specificity, PPV, NPV, and accuracy for 10 to 34-year-old patients was 66.7%, 100%, 100%, 40% and 72.7%, respectively. These measures were 90%, 100%, 100%, 83.3% and 93.3% for 35 to 69 year-old patients, respectively.

**Figure 1:**
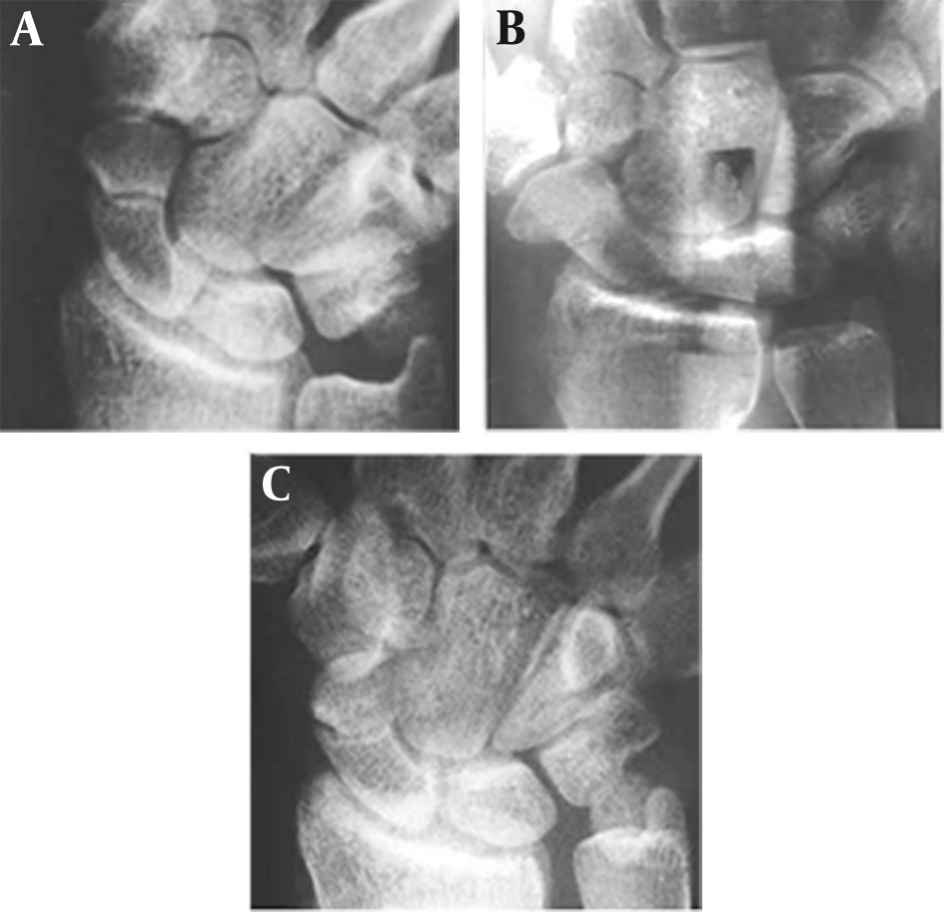
A 21-year-old man with wrist trauma. Conventional and panoramic radiographs were performed 10 weeks after injury because of the doubtful union of scaphoid fracture. A, Initial conventional radiograph shows doubtful scaphoid union. B, Panoramic radiograph shows the fracture partially united. C, Conventional radiograph obtained four months after trauma shows the fracture partially united, as seen in B.

**Figure 2:**
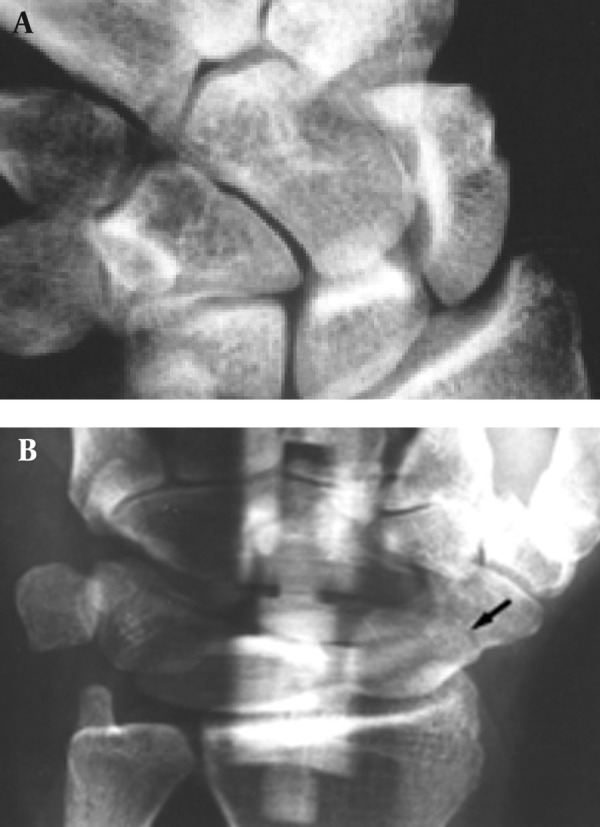
A 49-year-old woman with acute trauma of the wrist. Conventional and panoramic radiography were performed 1 week after injury. A, Conventional radiograph shows no evidence of fracture. B, Panoramic radiograph shows fracture line.

**Figure 3:**
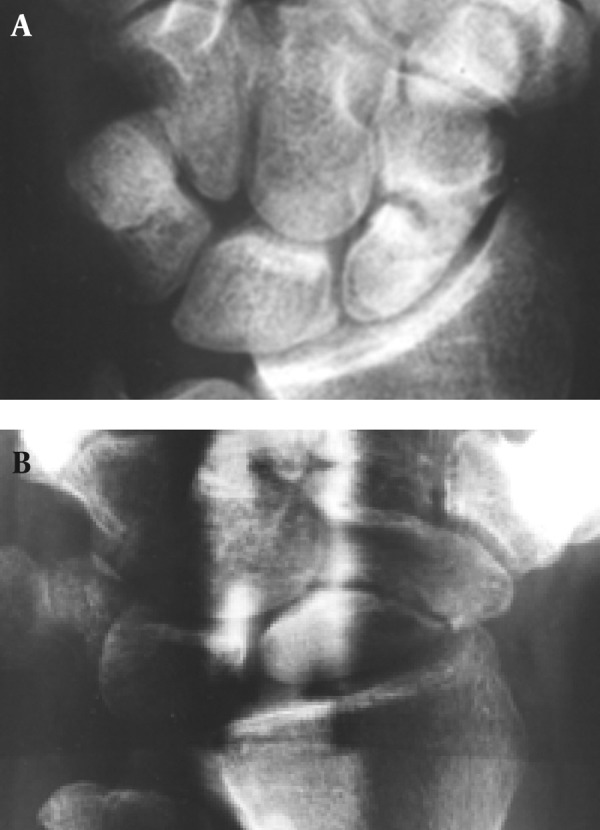
A 19-year-old man with wrist trauma. Conventional and panoramic radiography was performed 4 months after injury. A, Conventional radiograph shows doubtful union. B, Panoramic radiograph shows delayed union.

There was a significant relationship between the results of panoramic and final diagnosis in the diagnosis of scaphoid fracture union (P-value=0.015, P-value=0.001). Considering the high diagnostic value of panoramic radiography, it may be concluded that panoramic radiography can be used for the follow-up of healing of wrist scaphoid fractures in both age groups (Figures 1, 2, and 3).

The agreement of conventional and panoramic radiographies in diagnosis of fracture in the acute setting has been mentioned in table 5. In addition, the agreement of these two radiographies in the chronic setting for diagnosis of non healing has been mentioned in this table (Table 5).

**Table 5:** Agreement of Conventional and Panoramic Radiographies in Diagnosis of Fracture in Acute Setting and Non Healing in Chronic Setting

	Both Conventional and Panoramic Positive	Conventional Positive, Panoramic Negative	Conventional Negative, Panoramic Positive	Both Conventional and Panoramic Negative	Kappa [95% CI ^a^]	P-Value	Accuracy
Diagnosis of Fracture	62	32	21	7	-0.0795 [-0.24-0.08]	0.368	56.5%
Diagnosis of Non Healing	5	7	16	0	0.615 [0.21 -0.85]	<0.001	82.1%

Abbreviation: CI, Confidence interval

## 5. Discussion

Scaphoid fracture constitutes 2% of all fractures and 75% of wrist fractures (1, 14). Conventional radiographies are routinely used for evaluation of patients with clinical diagnosis of scaphoid fractures. Anyway, a considerable number of fractures are missed during primary examination due to difficulty in visualization of the fracture line, especially in cases without displacement. Daffner et al. (15) used oblique view of the wrist for these fractures with an angle of 30 degrees off the vertical axis towards the elbow. This results in elongation of the scaphoid bone and better visualization of the scaphoid in comparison to conventional radiography.

Dias et al. (16) stated that primary radiographies of the scaphoid bone in the second and third week after injury are not reliable. At present, disagreement persists over the merits of 2 weeks immobility in cases of probable clinical scaphoid fractures with negative findings in radiography. In order to identify hidden fractures of the scaphoid, other methods like computerized tomography (CT) or magnetic resonance imaging (MRI) (5-9) are used for early treatment or prevention of over treatment. The reason for not using these techniques is the high cost or unavailability. In a study by Houger et al (7), they concluded that high resolution ultrasonography is a correct and reliable method for evaluating hidden scaphoid fractures.

Panoramic radiography is used extensively for studying the dentomaxillofacial region. In addition, it has been used for studying the wrist (8, 9). Panoramic radiography of the wrist allows more detailed visualization of the scaphoid bone in comparison to conventional radiography and helps in determining the fracture line. The present study showed panoramic radiography of the wrist is better than conventional radiography for evaluating various clinical settings (suspected scaphoid fractures, scaphoid fractures or surgical injuries). Panoramic radiography ruled out the possibility of suspected scaphoid fractures in 74% of conventional radiographies. Panoramic radiography also showed 21.4% (12/56) of A2 type fractures without displacement according to the classification by Herbert and Fisher (17). Panoramic radiography also reveals most of the delayed healing fractures, unhealed fractures and healed fractures. A combination of clinical findings and panoramic radiographical findings that support diagnosis of fractures are worth consideration. The radiographs obtained in patients with flexibility restrictions of the wrist are not satisfactory as in immobile cases; the plaster cast has to be removed before panoramic radiography. Proper positioning of the wrist and immobility of the upper limb is essential for obtaining clear wrist images. In certain cases, there is blurring of the center of the image which is due to positioning of the appliance that an artifact creates on itself. In order to minimize the blurring, it is better to place the patient in the supine position.

The statistical findings of the present study showed a better understanding between clinical findings and panoramic radiography (90%) as compared to conventional radiography (53%). The intra- and interobserver agreement was also better for panoramic radiography. These findings showed that panoramic radiography is more reliable and accurate than conventional radiography in the diagnosis of scaphoid fractures. Therefore, panoramic radiography is indicated as a supplement in cases where conventional radiography has no results and can also help in the follow-up of scaphoid fractures. Radiographical algorithms have been proposed for the evaluation of possible acute scaphoid fractures, avascular necrosis, delayed healing or non-healing fractures. Panoramic radiography of the wrist is a simple, fast and economical X-ray that can detect or rule out a fracture. It is proposed that panoramic radiography should be used as a supplement to conventional radiography for clarity of radiographical studies without certainty.

Scaphoid fracture is the most common wrist fracture with an incidence of 46 fractures per 100 people per year (6). Scaphoid fractures are complicated as there is always a possibility of severity and chronicity (18-20). Even though there have been developments in the work-up with conventional radiography, two thirds of the scaphoid fractures remain hidden which is a challenge for radiologists (21, 22). In patients with hidden scaphoid fractures, associated changes are not significant enough to be diagnosed by conventional radiography (immediately after trauma). This is the case when they can be visualized after some time during follow up and therefore, these patients are treated for at least 10 days with an immobilizing plaster that results in unnecessary immobilization in 47% of the cases (22, 23). Correct and timely diagnosis can prevent over-treatment and decrease the complications; thus, decreasing health expenses significantly (4, 24, 25).

Berna et al. compared the diagnostic value of conventional and panoramic radiographies in cases of scaphoid fractures. They concluded that panoramic radiography is a useful technique for diagnosis and follow-up of scaphoid fractures and proposed its use as a supplement to conventional radiographies in patients with suspicious findings (26).

With respect to the results of the present study, it is proposed that panoramic radiography should also be prescribed along with conventional radiography for patients referred with wrist injury. In cases with suspected scaphoid fractures, only panoramic radiography should be prescribed because the patient is exposed to radiation only once in panoramic radiography, but in conventional radiography, the patient is exposed at least three to four times to radiation.
